# Dual Inhibition of Activin/Nodal/TGF-*β* and BMP Signaling Pathways by SB431542 and Dorsomorphin Induces Neuronal Differentiation of Human Adipose Derived Stem Cells

**DOI:** 10.1155/2016/1035374

**Published:** 2015-12-20

**Authors:** Vedavathi Madhu, Abhijit S. Dighe, Quanjun Cui, D. Nicole Deal

**Affiliations:** Orthopaedic Research Laboratories, Department of Orthopaedic Surgery, University of Virginia, Charlottesville, VA 22908, USA

## Abstract

Damage to the nervous system can cause devastating diseases or musculoskeletal dysfunctions and transplantation of progenitor stem cells can be an excellent treatment option in this regard. Preclinical studies demonstrate that untreated stem cells, unlike stem cells activated to differentiate into neuronal lineage, do not survive in the neuronal tissues. Conventional methods of inducing neuronal differentiation of stem cells are complex and expensive. We therefore sought to determine if a simple, one-step, and cost effective method, previously reported to induce neuronal differentiation of embryonic stem cells and induced-pluripotent stem cells, can be applied to adult stem cells. Indeed, dual inhibition of activin/nodal/TGF-*β* and BMP pathways using SB431542 and dorsomorphin, respectively, induced neuronal differentiation of human adipose derived stem cells (hADSCs) as evidenced by formation of neurite extensions, protein expression of neuron-specific gamma enolase, and mRNA expression of neuron-specific transcription factors Sox1 and Pax6 and matured neuronal marker NF200. This process correlated with enhanced phosphorylation of p38, Erk1/2, PI3K, and Akt1/3. Additionally, *in vitro* subcutaneous implants of SB431542 and dorsomorphin treated hADSCs displayed significantly higher expression of active-axonal-growth-specific marker GAP43. Our data offers novel insights into cell-based therapies for the nervous system repair.

## 1. Introduction

The human nervous system consists of the brain, spinal cord, autonomic nervous system (controlling involuntary functions such as heart rate, digestion, salivation, perspiration, urination, sexual arousal, and breathing), and peripheral nerves. Injuries and diseases of the nervous system have remained difficult challenges to clinicians and scientists all over the world.

Although Cajal's dogma that the neurons in the central nervous system (CNS) cannot regenerate has been refuted, it is recognized that the CNS lacks the ability to regenerate itself for the reestablishment of the correct axonal and dendritic connections [1]. Due to this inherent limitation, any damage to the CNS whether through neurodegenerative disease or trauma leads to devastating consequences such as Parkinson's disease, Alzheimer's diseases, or traumatic or ischemic brain injury. At present, no curative therapy is available for disease conditions of the CNS arising from loss of neurons or damaged axons and dendrites. Injury to peripheral nervous system (PNS) is relatively common, annually affecting more than a million people worldwide [2]. Unlike the CNS, the PNS possesses regeneration potential. Nerve injury can result from a nerve laceration or from avulsion of the nerve from its muscular insertion, both of which can result in fibrotic degeneration of the nerve and its motor unit due to loss of nerve signaling [3]. This can result in complete loss of muscle function, loss of limb function, and subsequent loss of work hours and diminished quality of life [4]. The current gold standards for nerve repair are suturing, nerve grafts, and neurotization if a nerve avulsion has occurred [5–8]. Each year over 50,000 peripheral nerve repair procedures are performed in the United States [9]. However, most patients treated with these techniques ultimately have poor muscle function because peripheral nerve regeneration is extremely slow (0.2 mm per day) and functional recovery is limited primarily by the progressive fall in regenerative capacity of the neurons with time and distance from their target muscles [10, 11]. Therefore, novel approaches that can effectively enhance repair of the CNS and PNS are needed.

Although in its infancy, cell-based therapy offers exciting potential [12–14]. Delivery of adult stem cells such as neural stem cells [15], bone marrow derived stem cells [16–19], and adipose derived stem cells [20] has shown encouraging outcomes for the treatment of Alzheimer's disease, Parkinson's disease, multiple sclerosis, spinal cord injury, Parry-Romberg syndrome, and Pelizaeus-Merzbacher disease, in animal models and humans. Local or intravenous delivery of stem cells isolated from a variety of adult tissue sources [21–39] has also been investigated using transected or crushed peripheral nerves (median, sciatic, facial, and cavernous) models in experimental animals. Use of embryonic stem cells (ESCs) [40] and induced-pluripotent stem cells (iPSCs) [41] has also been reported in the literature.

The transplantation of nontreated progenitor cells is not useful for regeneration of neural tissue because nontreated progenitor cells cannot differentiate to a neuron and cannot survive in the recipient's neural tissue [42]. It was reported that undifferentiated ADSCs did not survive till day 14 in a rat sciatic nerve defect model [43]. The success rate of stem cell mediated CNS or PNS repair is significantly higher when predifferentiated stem cells are used in comparison with undifferentiated stem cells [44, 45]. The Basso-Beattie-Bresnahan (BBB) score, indicator of recovery from spinal cord injury, was significantly higher in rats transplanted with neural-induced stem cells than in rats treated with undifferentiated stem cells and control untreated rats [44]. Another study comparing the use of differentiated and undifferentiated ADSCs for spinal cord repair revealed that the method of induction played an important role [45]. Since these methods of induction of neuronal differentiation are extremely complex and require multiple steps and expensive growth factors, it is necessary to develop newer and simpler protocols that are easy to use for the cell-based therapies of CNS or PNS repair and regeneration.

It is well known that activin/nodal signaling contributes to maintenance of pluripotency of hESCs. Inhibition of activin/nodal/TGF-*β* signaling leads to trophoblast differentiation similar to induction of trophoblast differentiation by BMP-4 [46]. Activin/nodal/TGF-*β* and BMP pathways naturally antagonize each other because they compete for a common signal transducer Smad4. Using this knowledge, investigators have recently shown that treatment of hESCs with activin/nodal/TGF-*β* inhibitor SB431542 for 8 days induces highly efficient and accelerated neural conversion [47]. Interestingly, addition of activin/nodal/TGF-*β* inhibitor SB431542 and natural BMP antagonist noggin synergistically induced rapid and complete neural conversion of >80% of hESCs as well as hiPSCs under adherent culture conditions [48]. Additionally, it was later reported that dorsomorphin which inhibits both activin/nodal/TGF-*β* and BMP pathways induced rapid and high-efficiency neural conversion in both hESCs and hiPSCs. Intriguingly, dorsomorphin was ineffective with mESCs [49]. It is also important to note that dorsomorphin is more specific for the BMP pathway.

This principle was confirmed by a report that simultaneous inhibition of both activin/nodal/TGF-*β* and BMP pathways with SB431542 and dorsomorphin, respectively, promoted significant neural differentiation from three hiPSCs and six hESCs lines showing marked variability in differentiation potential into specific lineages [50]. Similar findings were reported by other investigators using iPSC and hESC lines which further validated the concept [51, 52]. This easy and inexpensive approach of neuronal differentiation of stem cells has tremendous translational potential. However, ethical concerns associated with ESCs, technical difficulties in generating iPSCs, tumor forming ability of ESCs as well as iPSCs, and a recent report that iPSCs can induce immune response [53] are hindrances in their use for the nerve repair.

Therefore we investigated if inhibition of TGF and BMP signaling in hADSCs would induce neuronal differentiation. To our knowledge, this is the first time that hADSCs were induced to differentiate along a neuronal lineage using small molecule inhibitors of TGF-*β* and BMP signaling pathways. We found that this cost effective and single step method efficiently induced neuronal differentiation of hADSCs. Neuronally differentiated hADSCs have a vast number of applications in neurodegenerative diseases and enhancement of nerve injuries repair.

## 2. Materials and Methods

### 2.1. Cell Culture

hADSCs were purchased from Lonza (Lonza, Basel, Switzerland). The cells were maintained in basal medium (BM) which was Dulbecco's Modified Eagle's Medium (Gibco BRL, Gaithersburg, MD, USA) containing 10% fetal bovine serum (Hyclone Laboratories, Logan, Utah, USA), 50 mg/mL sodium ascorbate, 100 IU/mL penicillin G, and 100 mg/mL streptomycin in a humidified atmosphere of 5% carbon dioxide at 37°C.

All experiments were conducted with passage 8 (p-8) hADSCs at the starting cell density of 5000 cells/cm^2^.

To induce neuronal differentiation, hADSCs were grown in BM or BM supplemented with 2 *μ*M of BMP inhibitor dorsomorphin (DM) or 20 *μ*M of TGF-*β* inhibitor SB431542 (SB) (Sigma-Aldrich, St. Louis, MO, USA) or both for 7 and 14 days. To induce differentiation into Schwann cell phenotype using conventional method, hADSCs were grown in the nerve induction medium (NIM) in three consecutive steps: (1) hADSCs were treated with 1 mM *β*-mercaptoethanol in serum-free and ascorbate-free BM for 24 hours, (2) the cells were then grown in BM supplemented with 0.28 *μ*g/mL retinoic acid for 3 days, and (3) after retinoic acid treatment for 3 days the cells were transferred to BM supplemented with a mixture of growth factors 10 *μ*M forskolin (Sigma-Aldrich, St. Louis, MO, USA), 10 ng/mL basic FGF, 5 ng/mL PDGF (Life Technologies, Grand Island, NY, USA), and 200 ng/mL HRGb1 (R&D Systems, Minneapolis, MN, USA) and cultured for 10 days.

### 2.2. Real-Time PCR

Total RNA was extracted and purified using RNeasy mini kit (Qiagen, Valencia, CA, USA). RNA was reverse transcribed to cDNA using the iScript cDNA synthesis kit (Bio-Rad; Hercules, CA, USA) according to the manufacturer's protocol using random hexamer primers. The cDNA (100 ng total RNA equivalents) was mixed with iQ SYBR Green Supermix (Bio-Rad) and mRNA expression was determined using the iCycler iQTM (Bio-Rad) real-time PCR detection system and 18S rRNA gene expression as internal standard. The nerve specific primer sequences used in the real-time PCR reaction were as follows: Pax6 (F) 5′-GCCCTGGAGAAAGAGTTTGAGAGAACCCATT-3′, (R) 5′-GGGGAAATGAGTCCTGTTGAAGTGGTGC-3′, Sox1 (F) 5′-CACAACTCGGAGATCAGCAA-3′, (R) 5′-GTCCTTCTTGAGCAGCGTCT-3′, tubulin III*β* (F) 5′-GGAGATCGTGCACATCCAG-3′, (R) 5′-GAGGCCTCGTTGTAGTAGACG-3′, and NF200 (F) 5′-GAGGAACACCAAGTGGGAGA-3′, (R) 5′-CTTTGCTTCCTCCTTCGTTG-3′.

A measure of the mRNA for P0 (F) 5′-TGTGGTTTACACGGACAGGG-3′, (R) 5′-AGAGCAACAGCAGCAACAG-3′, p75NTR (F) 5′-TGGACAGCGTGACGTTCTCC-3′, (R) 5′-GATCTCCTCGCACTCGGCGT-3′, GFAP (F) 5′-GTCCATGTGGAGCTTGACG-3′, (R) 5′-CATTGAGCAGGTCCTGGTAC-3′, S100 (F) 5′-GGAAATCAAAGAGCAGGAGGT-3′, (R) 5′-ATTAGCTACAACACGGCTGGA-3′, and GAPDH (F) 5′-GAAGGTGAAGGTCGGAGT-3′, (R) 5′-CAAGCTTCCCGTTCTCAGC-3′ was determined using a cDNA template and gene specific primers by conventional PCR. The amplified DNA products from the PCR reactions were resolved in a 1% agarose gel and stained with ethidium bromide and densities of the bands were quantified using ImageJ software.

### 2.3. Western Blotting

The cells were grown in BM or BM supplemented with DM or SB or both for 7 and 14 days and then lysed with SDS sample buffer without bromophenol blue (125 mM Tris-HCl pH 6.8, 150 mM *β*-mercaptoethanol, 1% SDS, and 20% glycerol) in the presence of 1X protease inhibitor cocktail and 1 mM PMSF (Santa Cruz Biotechnology, Santa Cruz, CA, USA). The lysates were homogenized by ultrasonic homogenizer and then centrifuged at 20,000 rpm for 20 minutes at 4°C to remove the cell debris. The clear supernatants were transferred to a precooled fresh tube and immediately placed on ice. The protein concentration of the cell lysates was determined using a Bradford protein assay kit (Bio-Rad). 75 *μ*g of total proteins was resolved on 5–12% SDS-polyacrylamide gels at constant current of 80 V and electrotransferred to nitrocellulose membranes (Thermo Scientific, Waltham, MA, USA) at constant voltage of 10 V for overnight. The membranes were blocked with 5% BSA in TBST (50 mM Tris, pH 7.6, 150 mM NaCl, and 0.05% tween 20) for 1 hour at room temperature, washed, and incubated overnight at 4°C in 5% BSA in TBST containing specific antibody for the particular protein. The following antibodies were used: anti-phospho-Erk1/2 (1 : 300, BD Biosciences, San Jose, CA, USA), anti-phospho-Akt1/2/3 (1 : 1000, Santa Cruz Biotechnology, USA), anti-phospho-p38 (1 : 1000, Cell Signaling, Beverly, MA, USA), anti-phospho-PI3K (1 : 1000, Cell Signaling, USA), anti-phospho-Smad1/5/8 (1 : 1000, Cell Signaling, USA), anti-phospho-Smad2 (1 : 1000, Cell Signaling, USA), and anti-GAPDH (1 : 2000, Cell Signaling). The membranes were then incubated with HRP-conjugated secondary antibody (1 : 2000 in 5% nonfat dry milk in TBST) (Cell Singling) for 1 hour at room temperature followed by chemiluminescent substrate for HRP antibody (Thermo Scientific) and enhancer solution (Thermo Scientific) mixed in a 1 : 1 ratio. The membranes were incubated in the dark with CL-Xposure films (Pierce, Grand Island, NY, USA) and the films were developed to visualize the bands. To measure the band densities, electronic images were generated by placing the X-ray films in a GS-800 calibrated densitometer (Bio-Rad) and densities of the bands were quantified using ImageJ software.

The cells were plated in a 24-well plate and grown in BM or BM supplemented with DM or SB or both for 7 days. The cells were harvested after 7 days, washed with phosphate-buffered saline (PBS), fixed in 3.7% formaldehyde for 15 min, washed three times with PBS and permeabilized with 0.1% Triton X-100 for 2 min, and finally washed three times with PBS. After washing the cells, they were stained with anti-NSE (neuron-specific enolase) (1 : 50, Santa Cruz Biotechnology) or 1 *μ*g/mL Alexa Flour 488 conjugated phalloidin and DAPI (Sigma-Aldrich Corporation, St. Louis, MO, USA) for 1 hour and rinsed in PBS and incubated with an Alexa Fluor 594 conjugated goat anti-mouse secondary antibody (Life Technologies, Grand Island, NY, USA) for 40 min at room temperature. Following incubation, cells were washed extensively with PBS and then visualized under a microscope.

Frozen sections were treated with anti-GAP43 (1 : 100, Abcam, Cambridge, MA, USA) for 1 hr at 4°C. The sections were rinsed in PBS, incubated with an Alexa Fluor 594 conjugated goat anti-rabbit second antibody (Molecular Probes, Grand Island, NY, USA) for 40 min at room temperature, and observed under a fluorescent microscope. The sections were also stained with DAPI and density of red color was measured using ImageJ software.

### 2.4. Neuronal Differentiation of hADSCs* In Vivo*

Eight–ten-week-old C.B-17 SCID mice (Taconic, Germantown, NY, USA) were housed in the SPF Vivarium at the University of Virginia, which is fully accredited by the American Association for Accreditation of Laboratory Animal Care. This study was carried out in strict accordance with the recommendations in the Guide for the Care and Use of Laboratory Animals of the National Institutes of Health under Public Health Assurance number A3245-01. The protocol was approved by the University of Virginia Institutional Animal Care and Use Committee. The effect of* in vitro* stimulation of hADSCs on their potential to differentiate into neuronal lineage* in vivo* to induce axon growth was assessed using subcutaneous implantation of hADSCs in C.B-17 SCID mice [54]. The cells were grown in basal medium (BM) or BM supplemented with DM or SB or both for 1 week. After one week, cells were harvested, and 0.5 × 10^6^ cells were mixed with 200 *μ*L of Matrigel (BD Biosciences) at 4°C and kept chilled in a syringe until injected in the mice. C.B-17 SCID mice were anesthetized using intraperitoneal injection of ketamine (80 mg/kg) and xylazine (10 mg/kg). Using a syringe, the suspension of hADSCs in Matrigel was injected in the subcutaneous tissues of the mice. After 14 days of injection, the mice were sacrificed (*n* = 4), and implants were embedded in O.C.T. compound (Fisher Scientific, Waltham, MA, USA) and frozen in liquid nitrogen until staining.

### 2.5. Statistical Analysis

Data were presented as mean ± standard deviation from four different sample runs. We used SPSS 15.0 software for statistical analysis. ANOVA was performed. *p* value less than 0.05 was considered statistically significant.

## 3. Results

### 3.1. Inhibition of TGF-*β* and BMP Signaling Pathways Induces Neuronal Differentiation of hADSCs* In Vitro*

**Figure 1:**
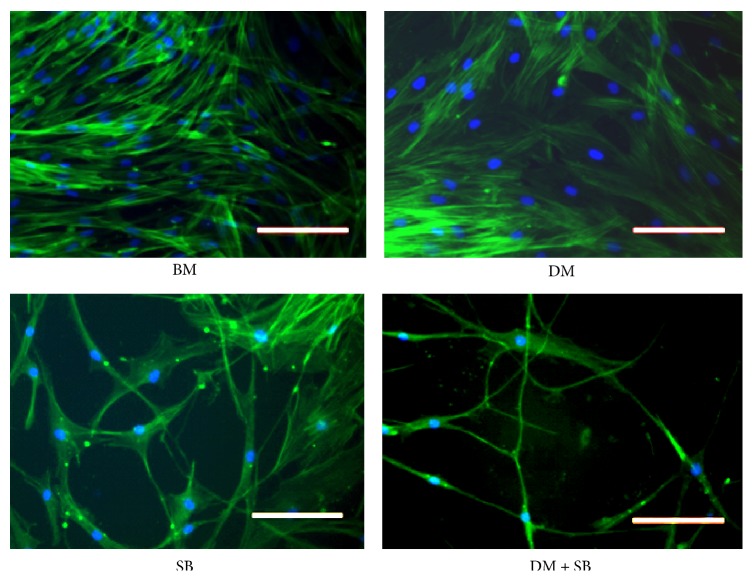
Human adipose derived stem cells (hADSCs) express neurite outgrowths upon dual inhibition of activin/nodal/TGF-*β* and BMP signaling pathways. hADSCs were cultured in basal medium DMEM containing 10% FBS (BM) or BM containing either activin/nodal/TGF-*β* pathway inhibitor SB431542 (SB) or BMP pathway inhibitor dorsomorphin (DM) or both for 14 days. Cells were stained with DAPI (staining nuclei) as well as FITC conjugated phalloidin (staining F-actin) and images were captured under a fluorescent microscope. Addition of SB or SB + DM induced neurite outgrowths in hADSCs. The length of neurite outgrowth in SB + DM group was significantly longer in comparison with other groups. Scale bar = 100 *μ*m.

To obtain morphological confirmation that inhibition of activin/nodal/TGF-*β* and BMP pathways induces neuronal differentiation of hADSCs, we compared the morphology of DM or SB or DM + SB treated cells with hADSCs cultured in control BM. Addition of DM + SB induced neurite outgrowths in hADSCs at day 14. Although addition of SB alone also induced neurite outgrowths, the maximum number of neurite outgrowths was observed in DM + SB group at day 14 (Figure 1). Neurite extension in hADSCs treated with DM and SB revealed that dual inhibition of activin/nodal/TGF-*β* and BMP signaling pathways induced axon sprouting in hADSCs.

**Figure 2:**
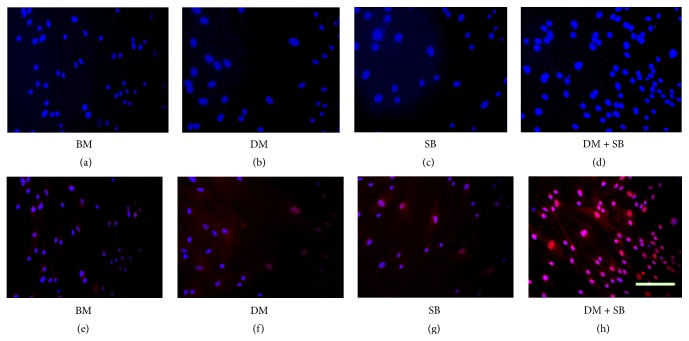
hADSCs express neuron-specific enolase upon dual inhibition of activin/nodal/TGF-*β* and BMP signaling pathways. hADSCs were cultured in BM or BM containing either activin/nodal/TGF-*β* pathway inhibitor SB or BMP pathway inhibitor DM or both for 14 days. Cells were stained with DAPI ((a)–(d)) or DAPI and anti-enolase antibody followed by Alexa Fluor 594 conjugated anti-rabbit secondary antibody ((e)–(h)). Images were captured under a fluorescent microscope. While addition of SB alone induced enolase expression in 14% of hADSCs, more than 85% of hADSCs expressed enolase upon addition of both SB and DM. Scale bar = 100 *μ*m.

The induction of a neuronal phenotype was further confirmed by the presence of neuron-specific enolase or gamma enolase at day 14 in the cells treated with both the inhibitors dorsomorphin and SB431542 (Figure 2). While no gamma enolase was detected in control hADSCs or hADSCs treated with DM, around 15% of the hADSCs treated with SB showed presence of gamma enolase in the nuclei (Figure 2). More than 85% of hADSCs treated with both SB and DM expressed gamma enolase.

**Figure 3:**
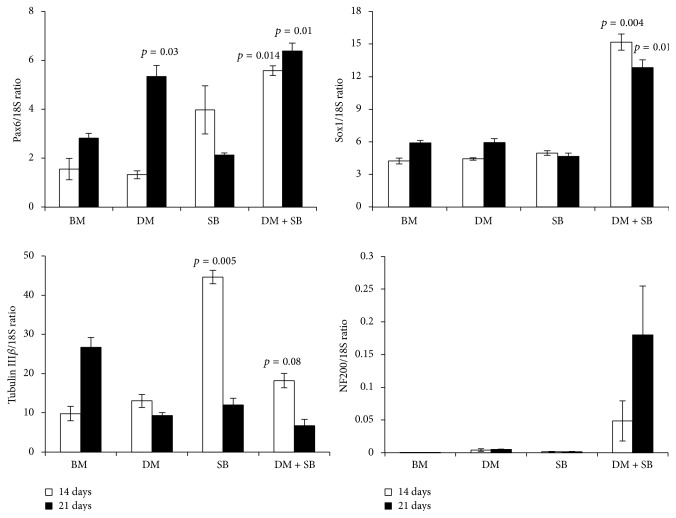
mRNA expression of neuronal markers in hADSCs upon dual inhibition of activin/nodal/TGF-*β* and BMP signaling pathways. hADSCs were cultured in BM or BM containing either activin/nodal/TGF-*β* pathway inhibitor SB or BMP pathway inhibitor DM or both for 14 and 21 days. The cells were harvested after 14 and 21 days to isolate RNA and prepare cDNA. Using gene specific primers and real-time PCR, expression of neuron cell specific transcription factors (Pax6 and Sox1), immature neuronal marker (tubulin III*β*), and matured neuronal marker (NF200) was quantified. *p* values are denoted compared to respective BM group.

The dual inhibition of activin/nodal/TGF-*β* and BMP signaling pathways promoted neuronal differentiation of hADSCs as evidenced by the significant increase in the expression of neural transcription factors Pax6 and Sox1 (Figure 3). Interestingly, inhibition of activin/nodal/TGF-*β* or BMP pathway alone was not sufficient to achieve enhancement of expression of Sox1 at days 14 and 21 but dual inhibition significantly enhanced Sox1 expression (Figure 3). Although inhibition of BMP pathway alone enhanced expression of Pax6 at day 21, a more robust increase in Pax6 expression was observed at day 21, only when TGF and BMP pathways both were inhibited (Figure 3). Moreover, inhibition of TGF and BMP pathways also significantly increased Pax6 expression at earlier time point (day 14) also which was not observed with inhibition of TGF or BMP pathway alone. This significant increase in expression of neural transcription factors Pax6 and Sox1 correlated with corresponding increase in expression of mature neuron cell markers NF200 and tubulin III*β* (Figure 3).

### 3.2. Dorsomorphin and SB431542 Induced Neuronal Differentiation of hADSCs Correlates with Activation of p38, Erk1/2, PI3K, and Akt Signaling and Inhibition of Smad Signaling Pathway

**Figure 4:**
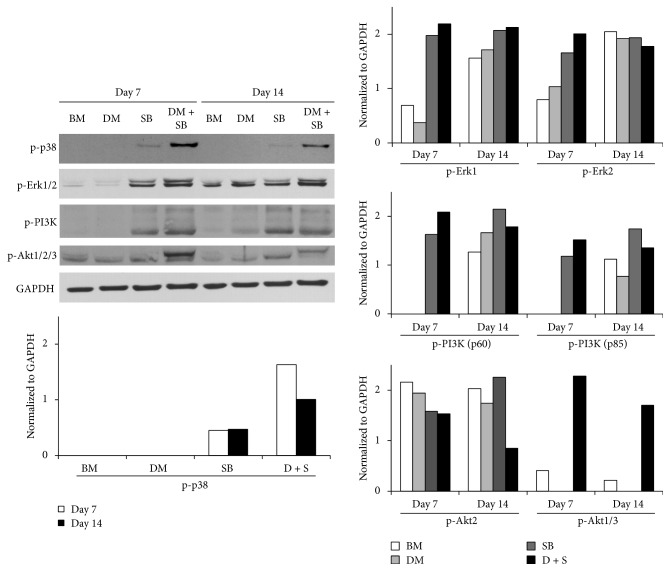
Dual inhibition of activin/nodal/TGF-*β* and BMP signaling pathways in hADSCs activates p38, Erk1/2, PI3K, and Akt1/2/3. hADSCs were grown in BM or BM supplemented with either activin/nodal/TGF-*β* pathway inhibitor SB or BMP pathway inhibitor DM or both for 7 and 14 days. The cells were harvested after 7 and 14 days and total proteins were harvested using extraction reagent. Proteins were resolved using electrophoresis and were transferred onto a nitrocellulose membrane using Trans-Blot cell. The bands were visualized by incubating the membrane with specific monoclonal antibodies followed by incubation with HRP-conjugated secondary antibody and then that with ECL reagent containing the substrate and enhancer solution. Intensity of bands was quantified using ImageJ software.

Since NGF induces neuronal differentiation of progenitor cells through Erk1/2 and PI3K/Akt signaling pathways [55] we measured phosphorylation of Erk, PI3K, and Akt in SB431542 and dorsomorphin treated hADSCs at days 7 and 14. We found that activation of these proteins was significantly enhanced in DM and SB treated hADSCs (Figure 4). Interestingly, we also found that dual inhibition of activin/nodal/TGF-*β* and BMP pathways in hADSCs significantly enhanced p38 activation (Figure 4). NGF withdrawal is reported to induce sustained activation of p38 and sustained inhibition of Erk in nerve progenitor cells [56]. A dynamic balance between growth factor-activated Erk and stress-activated p38 pathways plays an important role in determining whether a cell survives or undergoes apoptosis during neuronal development.

**Figure 5:**
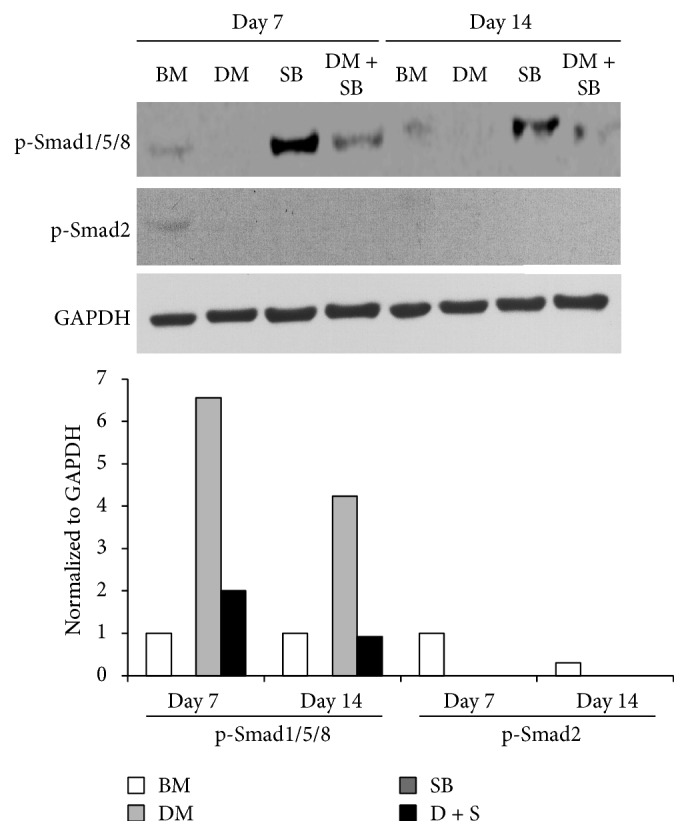
Dual inhibition of activin/nodal/TGF-*β* and BMP signaling pathways by DM and SB inhibits activation of Smad proteins in hADSCs. hADSCs were grown in BM or BM supplemented with either activin/nodal/TGF-*β* pathway inhibitor SB or BMP pathway inhibitor DM or both for 7 and 14 days. The cells were harvested after 7 and 14 days and total proteins were harvested using extraction reagent. Procedure was followed as mentioned in Figure 4.

We also found that SB treated hADSCs displayed increased activation of Smad1/5/8 while activation of Smad1/5/8 was significantly inhibited in hADSCs treated with DM and SB. Activation of Smad2 remained consistently inhibited in hADSCs treated with SB or DM or both (Figure 5). This inhibition of Smad1/5/8 and Smad2 correlated with the enhanced neuronal differentiation.

### 3.3. Nerve Induction Medium (NIM) Induces Schwann Cell Differentiation in hADSCs

**Figure 6:**
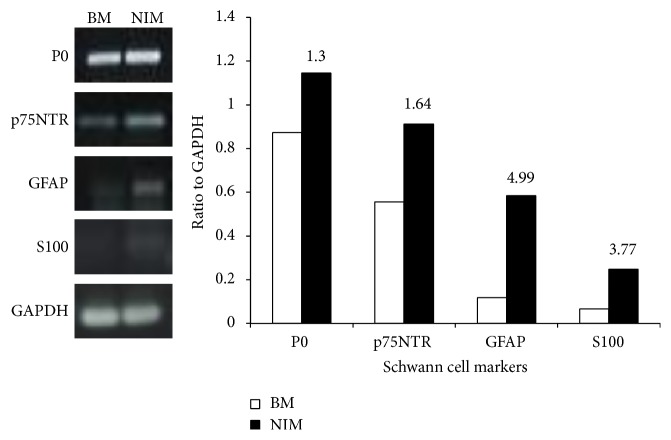
hADSCs can differentiate into Schwann cell phenotype. hADSCs were grown in BM and nerve induction medium (NIM) for 14 days. Using gene specific primers, mRNA expression of Schwann cell markers P0, p75NTR, GFAP, and S100 was determined from the cDNA templates. The PCR mixture was separated into a 1% agarose gel and the bands were visualized by ethidium bromide staining of the gels. The band intensities were quantified using ImageJ software.

mRNA expression of nerve-specific Schwann cell markers P0, p75NTR, GFAP, and S100 was significantly increased when hADSCs were grown in NIM in comparison with hADSCs cultured in BM (Figure 6). This data demonstrates that hADSCs can be induced to differentiate into both neuronal and Schwann cell-like phenotypes.

### 3.4. hADSCs Pretreated with SB431542 and Dorsomorphin Induce Nerve Fiber Growth upon Subcutaneous Implantation* In Vivo*

**Figure 7:**
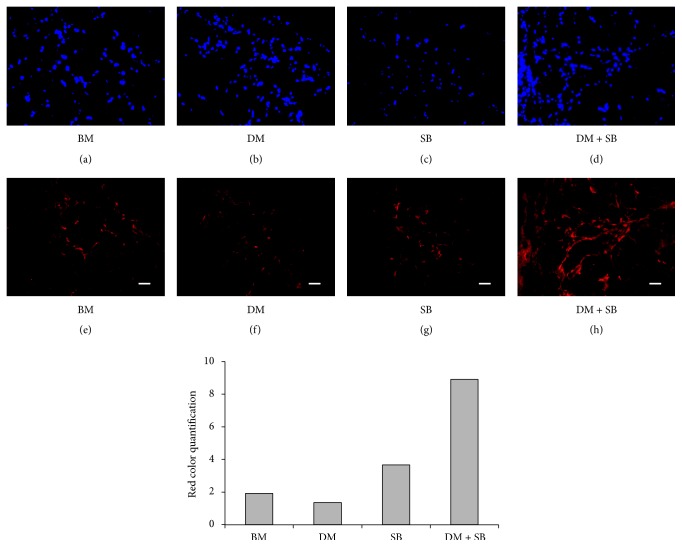
hADSCs activated with SB and DM for 7 days induce significant axonal outgrowth formation upon their transplantation* in vivo*. hADSCs were grown in BM or BM supplemented with either activin/nodal/TGF-*β* pathway inhibitor SB or BMP pathway inhibitor DM or both for 7 days. The cells were harvested after 7 days and 0.5 × 10^6^ cells were mixed with 200 *μ*L of Matrigel and implanted subcutaneously in C.B-17 SCID mice. Implants were harvested after 14 days and the cryosections were stained with DAPI ((a)–(d)) or with anti-GAP43 antibody followed by Alexa Fluor 596 conjugated secondary antibody ((e)–(h)). The slides were observed under a fluorescent microscope. Immunohistochemistry of hADSCs implants revealed that expression of axon growth cone specific GAP43 protein residing in the nerve fibers was maximum in implants of hADSCs treated with SB and DM. Scale bar = 100 *μ*m.

Since hADSCs treated with DM + SB exhibited enhanced neuronal differentiation* in vitro* we next determined whether hADSCs pretreated with DM and SB can undergo neuronal differentiation* in vivo*. hADSCs implanted subcutaneously after treatment with DM + SB had a significantly higher number of nerve fibers of significantly longer length in comparison with other groups (Figure 7). Expression of axon growth cone specific GAP43 protein residing in the nerve fiber was 8 times higher in SB and DM treated hADSCs as compared to the control group.

## 4. Discussion

Adipose derived stem cells (ADSCs) have several advantages over other adult stem cells. ADSCs can be isolated repeatedly in abundant numbers (500 times more than bone marrow derived stem cells) in a very easy and noninvasive procedure; they possess significant differentiation potential (osteogenic, adipogenic, myogenic, chondrogenic, and neurogenic lineages) proliferative capacity and resistance to senescence [57–62]. It is also reported that the transplantation effect of ADSCs is greater than BM-MSCs because they secrete more growth factors such as VEGF and HGF [63]. ADSCs are ideal alternative for enhancing nerve repair since they are known to produce nerve-growth promoting growth factors VEGF, bFGF, and HGF and neurotrophins BDNF, NGF, GDNF, and NT-1 [64–66].

Our study demonstrates that dual inhibition of activin/nodal/TGF-*β* and BMP pathways enhances neuronal differentiation of hADSCs* in vitro* (Figures 1, 2, and 3) and that hADSCs treated with activin/nodal/TGF-*β* inhibitor SB431542 and BMP inhibitor dorsomorphin for a week exhibited nerve sprouting upon transplantation in subcutaneous tissues of the C.B-17 SCID mice (Figure 7). Although the strategy of dual inhibition of activin/nodal/TGF-*β* and BMP pathways has been reported to induce neuronal differentiation of ESCs and iPSCs* in vitro* [40, 41] we demonstrated utility of this technique in ADSCs for the first time. In addition this is the first study to demonstrate that stem cells neuronally activated using this technique* in vitro* induce nerve sprouting* in vivo*.

Mechanistically, activin/nodal/TGF-*β* and BMP pathways inhibitors enhanced mRNA expression of two master regulators of the neural development: Pax6 and Sox1 (Figure 3). While Pax6 transcription factor is known to play a crucial role in the development of the central nervous system [67–70], Sox1 transcription factor plays a direct role in neural cell fate determination and differentiation [71]. We observed upregulation of Pax6 and Sox1 in SB431542 and dorsomorphin treated hADSCs; therefore we were curious to determine whether this upregulation leads to expression of immature neuronal marker *β*-tubulin 3 and mature neuronal marker neurofilament 200 (NF200) which indicates genuine neuronal differentiation. *β*-tubulin 3 is a structural protein expressed in the newly generated immature postmitotic neurons and differentiated neurons and mitotically active neuronal precursors [72], which contributes to microtubule stability in the neuronal cell bodies as well as in the axons and plays an important role in the axonal transport. NF200 filaments found in mature neurons are major components of the neuronal (axonal) cytoskeleton that provide structural support for axons and regulate axon diameter [73, 74] which in turn controls how fast electrical impulses travel down the axon [75]. While detectable levels of NF200 mRNA expression were observed only when the cells were treated with SB431542 and dorsomorphin, activin/nodal/TGF-*β* and BMP pathways played opposite roles in controlling expression of *β*-tubulin III (Figure 3). Inhibition of activin/nodal/TGF-*β* signaling alone significantly enhanced *β*-tubulin III expression but this enhancement required BMP signaling and the addition of BMP inhibitor reversed this effect in the DM + SB group (Figure 3).

Although we observed promising upregulation of Pax6, Sox1, and NF200 mRNA expression in the DM + SM group, mRNA expression of another mature neuronal marker *β*-tubulin III was not enhanced in the DM + SB group. However, the hypothesis that dual inhibition of activin/nodal/TGF-*β* and BMP signaling pathways enhances neuronal differentiation of hADSCs is substantiated by the fact that we observed significant increase in protein expression of another neuron-specific marker, enolase (NSE), also known as gamma enolase or enolase 2, in SB431542 and dorsomorphin treated hADSCs (Figure 2). NSE is a glycolytic enzyme expressed in mature neurons in both the central and the peripheral nervous systems [76]. NSE levels increase along with neuron maturation, reaching higher level with the morphological and functional maturation of neurons [77]. NSE not only is a specific marker for mature neurons but also is closely correlated with the differentiated neuronal state. The presence of significantly more neurite extensions in hADSCs treated with SB and DM validates this hypothesis (Figure 1).

After confirming the hypothesis* in vitro*, we sought to determine if hADSCs stimulated with SB431542 and dorsomorphin can differentiate into neural lineage upon transplantation* in vivo*. Naïve or noninduced stem cells rarely survive to differentiate into neural cells* in vivo*. Since survival of the transplanted cells is crucial for the therapeutic utilization, it is necessary to differentiate stem cells into their functional cell type prior to transplantation [13]. This strategy has been successfully utilized for the treatment of ischemic myocardium wherein mouse ADSCs were stimulated* in vitro* by NGF for 7 days and then transplanted* in vivo*. These mADSCs significantly enhanced regeneration by promoting the growth of the nerve sprouts and blood vessels in ischemic myocardium [78]. In another study, transplantation of mADSCs that were stimulated* in vitro* using similar method successfully induced functional recovery of crushed sensory and motor neurons [54]. A subcutaneous transplantation assay to determine ability of stem cells to induce sympathetic nerve sprouts ectopically has been previously described [54] and is used in this study. Although these studies demonstrated utility of ADSCs for nerve regeneration, the methods that were used for essential* in vitro* preconditioning of ADSCs required expensive NGF protein or viral vectors that may not be allowed by FDA for use in clinical setting. Moreover, ADSCs isolated from human tissues were not investigated. Our study demonstrated for the first time that transplantation of hADSCs that were neuronally differentiated* in vitro* using small molecule inhibitors which were inexpensive and safer than viral vectors significantly stimulated nerve sprouting* in vivo* (Figure 7). The numbers of growth associated protein 43 (GAP43) positive nerve fibers were significantly more in the implants of hADSCs treated with SB431542 and dorsomorphin in comparison with implants of untreated hADSCs or hADSCs treated with SB431542 or dorsomorphin alone. GAP43 is a phosphoprotein of the presynaptic membrane that plays a special role in synaptic reorganization. Importantly, in developing or regenerating neurons, GAP43 is an integral constituent of the growth cone [79–81]. Expression of GAP43 has been correlated with axon elongation in developing and regenerating neurons and GAP43 expression is considered an excellent marker of active axonal growth during development and nerve regeneration [82].

Since Erk and PI3K/Akt signaling regulate neuronal differentiation as well as neurite outgrowth, both essential events for neuronal regeneration after neuronal injury [83], we investigated the effect of SB431542 and dorsomorphin on Erk and PI3K/Akt activation in hADSCs. Inhibition of activin/nodal/TGF-*β* and BMP pathways activated Erk and PI3K/Akt signaling in hADSCs (Figure 4) indicating that SB431542 and dorsomorphin would promote neuronal differentiation of hADSCs. NGF is the most potent growth factor that induces neuronal differentiation of stem cells in an Erk and PI3K/Akt activation dependent manner [84–86]. NGF withdrawal is reported to induce sustained activation of p38 and sustained inhibition of Erks in nerve progenitor cells [56]. A dynamic balance between growth factor-activated Erk and stress-activated p38 pathways plays an important role in determining whether a cell survives or undergoes apoptosis during neuronal development. We observed that addition of SB431542 and dorsomorphin activated p38 in hADSCs (Figure 4). Further studies are required to determine role of p38 activation in dorsomorphin and SB431542 induced neuronal differentiation of hADSCs. We found that hADSCs treated with SB, which is a specific inhibitor of activin/nodal/TGF-*β* signaling pathway, displayed significantly increased activation of BMP signaling pathway as revealed by enhanced phosphorylation of Smad1/5/8. Addition of DM inhibited the activation of Smad1/5/8 (Figure 5). DM and SB efficiently inhibited activation of Smad2 (Figure 5). Dorsomorphin alone is reported to be sufficient to induce neural differentiation of hESCs and hiPSCs by inhibiting BMP as well as TGF-*β* signaling pathway since dorsomorphin is not a specific inhibitor of BMP pathway but it also inhibits activin/nodal/TGF-*β* signaling pathway [49]. However, in our study both dorsomorphin and SB431542 were required to induce neuronal differentiation of hADSCs. Our observation indicates that inhibiting activin/nodal/TGF-*β* and BMP signaling pathway simultaneously by DM and SB promotes neuronal differentiation and neurite outgrowth of hADSCs (Figure 5).

## 5. Conclusions

This study demonstrates that simultaneous inhibition of activin/nodal/TGF-*β* and BMP signaling pathways induces neuronal differentiation of hADSCs. This occurs through upregulation of expression of nerve transcription factors and modulation of various kinases which leads to neurite outgrowth* in vitro *and enhanced axon sprouting* in vivo*. To the best of our knowledge there is no other report in the literature demonstrating the use of small molecule inhibitors of activin/nodal/TGF-*β* and BMP signaling pathways to differentiate adult stem cells into neuronal lineage. Our data reveals the tremendous potential of dual inhibition of hADSCs for the treatment of neurological diseases and repair of the nervous system.

## References

[1] Ramon Y., Cajal S. (1928). *Degeneration and Regeneration of the Nervous System (Translated by RM Day from the 1913 Spanish edition)*.

[2] Martinez A. M., Goulart C. D., Ramalho B. D., Oliveira J. T., Almeida F. M. (2014). Neurotrauma and mesenchymal stem cells treatment: from experimental studies to clinical trials. *World Journal of Stem Cells*.

[3] Becker M., Lassner F., Fansa H., Mawrin C., Pallua N. (2002). Refinements in nerve to muscle neurotization. *Muscle & Nerve*.

[4] Bruyns C. N. P., Jaquet J.-B., Schreuders T. A. R., Kalmijn S., Kuypers P. D. L., Hovius S. E. R. (2003). Predictors for return to work in patients with median and ulnar nerve injuries. *Journal of Hand Surgery*.

[5] Menderes A., Yilmaz M., Vayvada H., Özer E., Barutçu A. (2002). Effects of nerve growth factor on the neurotization of denervated muscles. *Annals of Plastic Surgery*.

[6] Zhao C., Veltri K., Li S., Bain J. R., Fahnestock M. (2004). NGF, BDNF, NT-3, and GDNF mRNA expression in rat skeletal muscle following denervation and sensory protection. *Journal of Neurotrauma*.

[7] Iwata Y., Ozaki N., Hirata H. (2006). Fibroblast growth factor-2 enhances functional recovery of reinnervated muscle. *Muscle & Nerve*.

[8] Kang S.-B., Olson J. L., Atala A., Yoo J. J. (2012). Functional recovery of completely denervated muscle: implications for innervation of tissue-engineered muscle. *Tissue Engineering Part A*.

[10] Wolford L. M., Stevao E. L. L. (2003). Considerations in nerve repair. *Baylor University Medical Center Proceedings*.

[11] Gordon T., Tyreman N., Raji M. A. (2011). The basis for diminished functional recovery after delayed peripheral nerve repair. *Journal of Neuroscience*.

[12] Kalladka D., Muir K. W. (2014). Brain repair: cell therapy in stroke. *Stem Cells and Cloning: Advances and Applications*.

[13] Kanno H. (2013). Regenerative therapy for neuronal diseases with transplantation of somatic stem cells. *World Journal of Stem Cells*.

[14] Forostyak S., Jendelova P., Sykova E. (2013). The role of mesenchymal stromal cells in spinal cord injury, regenerative medicine and possible clinical applications. *Biochimie*.

[15] Gupta N., Henry R. G., Strober J. (2012). Neural stem cell engraftment and myelination in the human brain. *Science Translational Medicine*.

[16] Lee J. K., Jin H. K., Endo S., Schuchman E. H., Carter J. E., Bae J.-S. (2010). Intracerebral transplantation of bone marrow-derived mesenchymal stem cells reduces amyloid-beta deposition and rescues memory deficits in Alzheimer's disease mice by modulation of immune responses. *Stem Cells*.

[17] Venkataramana N. K., Kumar S. K. V., Balaraju S. (2010). Open-labeled study of unilateral autologous bone-marrow-derived mesenchymal stem cell transplantation in Parkinson's disease. *Translational Research*.

[18] Freedman M. S., Bar-Or A., Atkins H. L. (2010). The therapeutic potential of mesenchymal stem cell transplantation as a treatment for multiple sclerosis: consensus report of the international MSCT study group. *Multiple Sclerosis*.

[19] Park J. H., Kim D. Y., Sung I. Y. (2012). Long-term results of spinal cord injury therapy using mesenchymal stem cells derived from bone marrow in humans. *Neurosurgery*.

[20] Koh K. S., Oh T. S., Kim H. (2012). Clinical application of human adipose tissue-derived mesenchymal stem cells in progressive hemifacial atrophy (Parry-Romberg disease) with microfat grafting techniques using 3-dimensional computed tomography and 3-dimensional camera. *Annals of Plastic Surgery*.

[21] Xu L., Zhou S., Feng G.-Y. (2012). Neural stem cells enhance nerve regeneration after sciatic nerve injury in rats. *Molecular Neurobiology*.

[22] Oliveira J. T., Almeida F. M., Biancalana A. (2010). Mesenchymal stem cells in a polycaprolactone conduit enhance median-nerve regeneration, prevent decrease of creatine phosphokinase levels in muscle, and improve functional recovery in mice. *Neuroscience*.

[23] Frattini F., Pereira Lopes F. R., Almeida F. M. (2012). Mesenchymal stem cells in a polycaprolactone conduit promote sciatic nerve regeneration and sensory neuron survival after nerve injury. *Tissue Engineering—Part A*.

[24] Costa H. J. Z. R., Ferreira Bento R., Salomone R. (2013). Mesenchymal bone marrow stem cells within polyglycolic acid tube observed in vivo after six weeks enhance facial nerve regeneration. *Brain Research*.

[25] Dadon-Nachum M., Sadan O., Srugo I., Melamed E., Offen D. (2011). Differentiated mesenchymal stem cells for sciatic nerve injury. *Stem Cell Reviews and Reports*.

[26] You D., Jang M. J., Lee J. (2013). Periprostatic implantation of human bone marrow-derived mesenchymal stem cells potentiates recovery of erectile function by intracavernosal injection in a rat model of cavernous nerve injury. *Urology*.

[27] Marconi S., Castiglione G., Turano E. (2012). Human adipose-derived mesenchymal stem cells systemically injected promote peripheral nerve regeneration in the mouse model of sciatic crush. *Tissue Engineering Part A*.

[28] Zhang Y., Luo H., Zhang Z. (2010). A nerve graft constructed with xenogeneic acellular nerve matrix and autologous adipose-derived mesenchymal stem cells. *Biomaterials*.

[29] Wang Y., Zhao Z., Ren Z. (2012). Recellularized nerve allografts with differentiated mesenchymal stem cells promote peripheral nerve regeneration. *Neuroscience Letters*.

[30] Carriel V., Garrido-Gómez J., Hernández-Cortés P. (2013). Combination of fibrin-agarose hydrogels and adipose-derived mesenchymal stem cells for peripheral nerve regeneration. *Journal of Neural Engineering*.

[31] Ghoreishian M., Rezaei M., Beni B. H., Javanmard S. H., Attar B. M., Zalzali H. (2013). Facial nerve repair with Gore-Tex tube and adipose-derived stem cells: an animal study in dogs. *Journal of Oral and Maxillofacial Surgery*.

[32] Lin H., Liu F., Zhang C. (2009). Human amniotic fluid mesenchymal stem cells in combination with hyperbaric oxygen augument peripheral nerve regeneration. *Molecular Neurobiology*.

[33] Cheng F.-C., Tai M.-H., Sheu M.-L. (2010). Enhancement of regeneration with glia cell line-derived neurotrophic factor-transduced human amniotic fluid mesenchymal stem cells after sciatic nerve crush injury. *Journal of Neurosurgery*.

[34] Matsuse D., Kitada M., Kohama M. (2010). Human umbilical cord-derived mesenchymal stromal cells differentiate into functional Schwann cells that sustain peripheral nerve regeneration. *Journal of Neuropathology and Experimental Neurology*.

[35] Gärtner A., Pereira T., Alves M. G. (2012). Use of poly(DL-lactide-*ε*-caprolactone) membranes and mesenchymal stem cells from the Wharton's jelly of the umbilical cord for promoting nerve regeneration in axonotmesis: *in vitro* and *in vivo* analysis. *Differentiation*.

[36] Amoh Y., Li L., Campillo R. (2005). Implanted hair follicle stem cells form Schwann cells that support repair of severed peripheral nerves. *Proceedings of the National Academy of Sciences of the United States of America*.

[37] Pan H. C., Chin C. S., Yang D. Y. (2009). Human amniotic fluid mesenchymal stem cells in combination with hyperbaric oxygen augment peripheral nerve regeneration. *Neurochemical Research*.

[38] Amoh Y., Hamada Y., Aki R., Kawahara K., Hoffman R. M., Katsuoka K. (2010). Direct transplantation of uncultured hair-follicle Pluripotent Stem (hfPS) cells promotes the recovery of peripheral nerve injury. *Journal of Cellular Biochemistry*.

[39] Park B.-W., Kang D.-H., Kang E.-J. (2012). Peripheral nerve regeneration using autologous porcine skin-derived mesenchymal stem cells. *Journal of Tissue Engineering and Regenerative Medicine*.

[40] Cui L., Jiang J., Wei L. (2008). Transplantation of embryonic stem cells improves nerve repair and functional recovery after severe sciatic nerve axotomy in rats. *Stem Cells*.

[41] Uemura T., Takamatsu K., Ikeda M. (2012). Transplantation of induced pluripotent stem cell-derived neurospheres for peripheral nerve repair. *Biochemical and Biophysical Research Communications*.

[42] Yamada H., Dezawa M., Shimazu S. (2003). Transfer of the von Hippel-Lindau gene to neuronal progenitor cells in treatment for Parkinson's disease. *Annals of Neurology*.

[43] Erba P., Mantovani C., Kalbermatten D. F., Pierer G., Terenghi G., Kingham P. J. (2010). Regeneration potential and survival of transplanted undifferentiated adipose tissue-derived stem cells in peripheral nerve conduits. *Journal of Plastic, Reconstructive and Aesthetic Surgery*.

[44] Pedram M. S., Dehghan M. M., Soleimani M., Sharifi D., Marjanmehr S. H., Nasiri Z. (2010). Transplantation of a combination of autologous neural differentiated and undifferentiated mesenchymal stem cells into injured spinal cord of rats. *Spinal Cord*.

[45] Zhang H.-T., Luo J., Sui L.-S. (2009). Effects of differentiated versus undifferentiated adipose tissue-derived stromal cell grafts on functional recovery after spinal cord contusion. *Cellular and Molecular Neurobiology*.

[46] Wu Z., Zhang W., Chen G. (2008). Combinatorial signals of activin/nodal and bone morphogenic protein regulate the early lineage segregation of human embryonic stem cells. *The Journal of Biological Chemistry*.

[47] Patani R., Compston A., Puddifoot C. A. (2009). Activin/nodal inhibition alone accelerates highly efficient neural conversion from human embryonic stem cells and imposes a caudal positional identity. *PLoS ONE*.

[48] Chambers S. M., Fasano C. A., Papapetrou E. P., Tomishima M., Sadelain M., Studer L. (2009). Highly efficient neural conversion of human ES and iPS cells by dual inhibition of SMAD signaling. *Nature Biotechnology*.

[49] Zhou J., Su P., Li D., Tsang S., Duan E., Wang F. (2010). High-efficiency induction of neural conversion in human ESCs and human induced pluripotent stem cells with a single chemical inhibitor of transforming growth factor beta superfamily receptors. *Stem Cells*.

[50] Kim D.-S., Lee J. S., Leem J. W. (2010). Robust enhancement of neural differentiation from human ES and iPS cells regardless of their innate difference in differentiation propensity. *Stem Cell Reviews and Reports*.

[51] Morizane A., Doi D., Kikuchi T., Nishimura K., Takahashi J. (2011). Small-molecule inhibitors of bone morphogenic protein and activin/nodal signals promote highly efficient neural induction from human pluripotent stem cells. *Journal of Neuroscience Research*.

[52] Surmacz B., Fox H., Gutteridge A., Fish P., Lubitz S., Whiting P. (2012). Directing differentiation of human embryonic stem cells toward anterior neural ectoderm using small molecules. *Stem Cells*.

[53] Zhao T., Zhang Z.-N., Rong Z., Xu Y. (2011). Immunogenicity of induced pluripotent stem cells. *Nature*.

[54] Lopatina T., Kalinina N., Karagyaur M. (2011). Adipose-derived stem cells stimulate regeneration of peripheral nerves: BDNF secreted by these cells promotes nerve healing and axon growth *De Novo*. *PLoS ONE*.

[55] Sofroniew M. V., Howe C. L., Mobley W. C. (2001). Nerve growth factor signaling, neuroprotection, and neural repair. *Annual Review of Neuroscience*.

[56] Xia Z., Dickens M., Raingeaud J., Davis R. J., Greenberg M. E. (1995). Opposing effects of ERK and JNK-p38 MAP kinases on apoptosis. *Science*.

[57] Gimble J. M., Katz A. J., Bunnell B. A. (2007). Adipose-derived stem cells for regenerative medicine. *Circulation Research*.

[58] Kern S., Eichler H., Stoeve J., Klüter H., Bieback K. (2006). Comparative analysis of mesenchymal stem cells from bone marrow, umbilical cord blood, or adipose tissue. *Stem Cells*.

[59] Izadpanah R., Trygg C., Patel B. (2006). Biologic properties of mesenchymal stem cells derived from bone marrow and adipose tissue. *Journal of Cellular Biochemistry*.

[60] Dmitrieva R. I., Minullina R., Bilibina A. A., Tarasova O. V., Anisimov S. V., Zaritskey A. Y. (2012). Bone marrow- and subcutaneous adipose tissue-derived mesenchymal stem cells: differences and similarities. *Cell Cycle*.

[61] Hass R., Kasper C., Böhm S., Jacobs R. (2011). Different populations and sources of human mesenchymal stem cells (MSC): a comparison of adult and neonatal tissue-derived MSC. *Cell Communication and Signaling*.

[62] Fraser J. K., Wulur I., Alfonso Z., Hedrick M. H. (2006). Fat tissue: an underappreciated source of stem cells for biotechnology. *Trends in Biotechnology*.

[63] Ikegame Y., Yamashita K., Hayashi S.-I. (2011). Comparison of mesenchymal stem cells from adipose tissue and bone marrow for ischemic stroke therapy. *Cytotherapy*.

[64] Wei X., Du Z., Zhao L. (2009). IFATS collection: the conditioned media of adipose stromal cells protect against hypoxia-ischemia-induced brain damage in neonatal rats. *Stem Cells*.

[65] Rehman J., Traktuev D., Li J. (2004). Secretion of angiogenic and antiapoptotic factors by human adipose stromal cells. *Circulation*.

[66] Wei X., Zhao L., Zhong J. (2009). Adipose stromal cells-secreted neuroprotective media against neuronal apoptosis. *Neuroscience Letters*.

[67] Zhang X., Huang C. T., Chen J. (2010). Pax6 is a human neuroectoderm cell fate determinant. *Cell Stem Cell*.

[68] Vincent M.-C., Pujo A.-L., Olivier D., Calvas P. (2003). Screening for PAX6 gene mutations is consistent with haploinsufficiency as the main mechanism leading to various ocular defects. *European Journal of Human Genetics*.

[69] Mo Z., Zecevic N. (2008). Is Pax6 critical for neurogenesis in the human fetal brain?. *Cerebral Cortex*.

[70] Walcher T., Xie Q., Sun J. (2013). Functional dissection of the paired domain of Pax6 reveals molecular mechanisms of coordinating neurogenesis and proliferation. *Development*.

[71] Pevny L. H., Sockanathan S., Placzek M., Lovell-Badge R. (1998). A role for SOX1 in neural determination. *Development*.

[72] Halbach O. V. B. U. (2007). Immunohistological markers for staging neurogenesis in adult hippocampus. *Cell and Tissue Research*.

[73] Portier M.-M., Escurat M., Landon F., Djabali K., Bousquet O. (1993). Peripherin and neurofilaments: expression and role during neural development. *Comptes Rendus de l'Academie des Sciences—Series III*.

[74] Iwanaga T., Takahashi Y., Fujita T. (1989). Immunohistochemistry of neuron-specific and glia-specific proteins.. *Archives of Histology and Cytology*.

[75] Alberts B., Johnson A., Lewis J., Raff M., Roberts K., Walter P. (2002). *Molecular Biology of the Cell*.

[76] Schmechell D. E., Marangos P. J., Zis A. P., Brightman M., Goodwin F. K. (1978). Brain endolases as specific markers of neuronal and glial cells. *Science*.

[77] Marangos P. J., Schmechel D. E., Parma A. M., Goodwin F. K. (1980). Developmental profile of neuron-specific (NSE) and non-neuronal (NNE) enolase. *Brain Research*.

[78] Cai L., Johnstone B. H., Cook T. G. (2009). IFATS collection: human adipose tissue-derived stem cells induce angiogenesis and nerve sprouting following myocardial infarction, in conjunction with potent preservation of cardiac function. *Stem Cells*.

[79] Meiri K. F., Pfenninger K. H., Willard M. B. (1986). Growth-associated protein, GAP-43, a polypeptide that is induced when neurons extend axons, is a component of growth cones and corresponds to pp46, a major polypeptide of a subcellular fraction enriched in growth cones. *Proceedings of the National Academy of Sciences of the United States of America*.

[80] Skene J. H. P., Jacobson R. D., Snipes G. J., McGuire C. B., Norden J. J., Freeman J. A. (1986). A protein induced during nerve growth (GAP-43) is a major component of growth-cone membranes. *Science*.

[81] Smith C. L., Afroz R., Bassell G. J., Furneaux H. M., Perrone-Bizzozero N. I., Burry R. W. (2004). GAP-43 mRNA in growth cones is associated with HuD and ribosomes. *Journal of Neurobiology*.

[82] Skene J. H. P., Willard M. (1981). Axonally transported proteins associated with axon growth in rabbit central and peripheral nervous systems. *Journal of Cell Biology*.

[83] Wang X., Wang Z., Yao Y. (2011). Essential role of ERK activation in neurite outgrowth induced by *α*-lipoic acid. *Biochimica et Biophysica Acta—Molecular Cell Research*.

[84] Meng X.-L., Rennert O. M., Chan W.-Y. (2007). Human chorionic gonadotropin induces neuronal differentiation of PC12 cells through activation of stably expressed lutropin/choriogonadotropin receptor. *Endocrinology*.

[85] Obara Y., Yamauchi A., Takehara S. (2009). ERK5 activity is required for nerve growth factor-induced neurite outgrowth and stabilization of tyrosine hydroxylase in PC12 cells. *The Journal of Biological Chemistry*.

[86] Washio A., Kitamura C., Jimi E., Terashita M., Nishihara T. (2009). Mechanisms involved in suppression of NGF-induced neuronal differentiation of PC12 cells by hyaluronic acid. *Experimental Cell Research*.

